# Molecular Targeted Therapies in Metastatic Prostate Cancer: Recent Advances and Future Challenges

**DOI:** 10.3390/cancers15112885

**Published:** 2023-05-23

**Authors:** Carlo Sorrentino, Emma Di Carlo

**Affiliations:** 1 Department of Medicine and Sciences of Aging, “G. d’Annunzio” University of Chieti-Pescara, 66100 Chieti, Italy; edicarlo@unich.it; 2 Anatomic Pathology and Immuno-Oncology Unit, Center for Advanced Studies and Technology (CAST), “G. d’Annunzio” University of Chieti-Pescara, 66100 Chieti, Italy

**Keywords:** anti-angiogenic therapy, castration-resistant prostate cancer, immunotherapy, metastasis, molecular targeted therapies

## Abstract

**Simple Summary:**

Recent studies have highlighted the importance of molecular targeted therapies in metastatic castration-resistant prostate cancer. These therapies, which aim to block specific molecules and related pathways in cancer cells, or in cells present in their microenvironment, and inhibit their proliferation, invasiveness and migration, while minimizing damage to healthy tissues, can be distinguished into four main categories: Prostate-Specific Membrane Antigen-targeted radionuclide therapies; DNA repair inhibitors; therapies targeting tumor neovascularization; and immune checkpoint inhibitors. The purpose of this review is to illustrate the characteristics, efficacy and limitations, of these therapies, some of which are already approved for clinical use while others are in clinical trials or at the pre-clinical stage of development, and to explore future research perspectives. Different solutions, from combined treatment with traditional drugs, to the use of nanomedicine for the selective release at the the tumor site, will be discussed, in order to improve their tolerability and therapeutic efficacy.

**Abstract:**

Prostate cancer is the most frequent malignant tumor in men, and, despite the great improvements in survival in patients with localized cancer, the prognosis for metastatic disease remains poor. Novel molecular targeted therapies, which block specific molecules or signaling pathways in tumor cells or in their microenvironment, have shown encouraging results in metastatic castration-resistant prostate cancer. Among these therapeutic approaches, prostate-specific membrane antigen-targeted radionuclide therapies and DNA repair inhibitors represent the most promising ones, with some therapeutic protocols already approved by the FDA, whereas therapies targeting tumor neovascularization and immune checkpoint inhibitors have not yet demonstrated clear clinical benefits. In this review, the most relevant studies and clinical trials on this topic are illustrated and discussed, together with future research directions and challenges.

## 1. Introduction

Prostate cancer is the second most frequent cancer in males (15.1%) and the fifth most frequent cause of cancer-related death (6.83%) in men worldwide [1]. In Europe, it is the most frequent malignant tumor (22.2%) and the third cause of cancer-related death (10.0%) in men, after lung and colorectal cancers [1]. 

Although the traditional treatment approach, which includes surgical resection, radiotherapy, and hormone therapy [2], has led to great improvements in both survival and quality of life in patients with localized disease (5-year survival rate of nearly 100%), the prognosis for metastatic disease remains poor, with a 5-year survival rate of only 30% [3].

Since ~65% of new prostate cancers are diagnosed in males over 65 years old and in 25% of males over 75 years old [4], the incidence of prostate cancer and related mortality are expected to rise due to an increase in life expectancy and the aging population. Therefore, there is a critical need for the development of innovative and tolerable therapeutic approaches, which are effective in the treatment of advanced disease and suitable for frail elderly patients.

In recent years, molecular targeted therapies, aimed at blocking specific molecules or signaling pathways in tumor cells or in their microenvironment, with a low risk of damage to normal tissues, have demonstrated their efficacy in several types of cancer (Table 1) [5]. Various trials have had encouraging results in the treatment of metastatic prostate cancer [6]. The molecular targeted therapies actually under study, or that have entered clinical use or trials for the treatment of advanced prostate cancer (Table 2 and Table 3), are described below.

## 2. Molecular Targeted Therapies for the Treatment of Metastatic Prostate Cancer 

**Figure 1:**
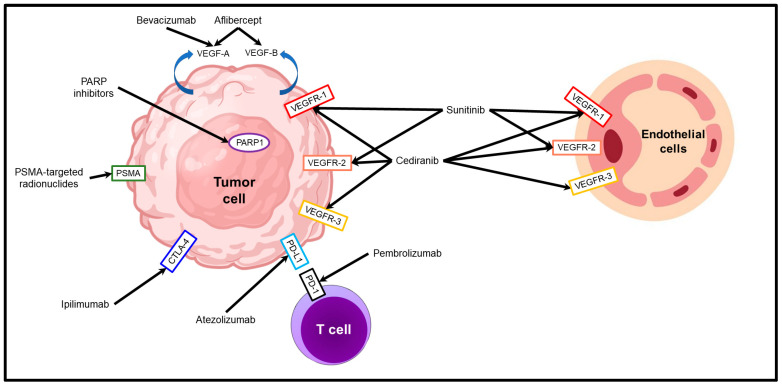
Molecular targeted therapies in prostate cancer. The molecular targets and the mechanism of action of PSMA-targeted radionuclides, DNA repair inhibitors, anti-angiogenic factors (aflibercept, bevacizumab, cediranib, and sunitinib) and immune checkpoint inhibitors (atezolimumab, ipilimumab, and pembrolizumab) are illustrated.

Molecular targeted therapies, approved or under study for the treatment of metastatic prostate cancer, belong to one of four categories: prostate-specific membrane antigen (PSMA)-targeted radionuclide therapies; DNA repair inhibitors; therapies targeting tumor neovascularization; or immune checkpoint inhibitors (Figure 1).

### 2.1. PSMA-Targeted Radionuclide Therapies

Radioligand therapy (RLT) has gained great interest in the last few years as cancer treatment has become more specific and personalized.

In contrast to external-beam radiation therapy, RLT targets cancer cells and their microenvironment, while sparing normal cells [7]. RLT delivers radioactive emitters directly to tumor-associated targets. This therapeutic approach provides several advantages over existing therapeutic modalities. Unlike traditional radiotherapy, in which the radiation from an external beam is delivered systemically and cannot be focused on the tumor, in RLT, the radiation is delivered selectively to cancer cells using a carrier that binds to a cancer specific target. Once there, the radioactive atoms cause DNA damage specifically in the tumor, thus inducing cancer cell death. The use of a cancer-targeted delivery system and the limited range of action of the radioactive alpha and beta emitters provide clinicians with a potentially very high therapeutic index [7].

In the past few decades, bone-targeted radionuclides, strontium-89 and samarium-153, have been used to treat chronic pain due to bone metastases [8,9]. After intravenous injection, these radionuclides selectively localize in osteoblastic prostate cancer metastases, but not in lymph node and visceral metastases, resulting in a lack of survival benefit.

In the last decade, the PSMA protein has been the focus of many studies concerning prostate cancer, since it is highly expressed in prostate cancer cells compared to normal epithelial cells, and its expression increases in the advanced stages of the disease [10].

To date, the only PSMA-based RLT agent approved for clinical use in patients with metastatic prostate cancer, on the basis of the results obtained from the phase III VISION trial (NCT03511664) [11], is the ^177^Lu-PSMA-617, in which the beta emitter lutetium-177 is linked to PSMA-617 (or vipivotide tetraxetan), a highly specific PSMA ligand.

In the VISION study, 551 patients from 84 sites across North America and Europe with PSMA-positive metastatic castration-resistant prostate cancer (mCRPC), and whose cancer progressed despite treatment with androgen receptor inhibitors and taxane chemotherapy, were treated with four to six cycles of ^177^Lu-PSMA-617 every 6 weeks, while 280 patients with the same clinic-pathological characteristics were treated with standard-of-care therapy (control group). In the experimental group, 9.2% of the patients showed a complete response, which was absent in the control group, and 41.8% showed a partial response compared to 3% of the control group. Furthermore, the median overall survival was significantly longer in the experimental group (15.3 months) in comparison to the control group (11.3 months) [hazard ratio (HR), 0.62; 95% confidence interval (CI), 0.52–0.74; *p* < 0.001]. Fatigue, dry mouth, anemia, and back pain were the most common, but well tolerated, side effects.

However, many questions remain unanswered, such as the optimal dose and schedule of ^177^Lu-PSMA-617 infusions, the optimal selection criteria of patients, the efficacy in combination with other therapies, and the long-term safety. To address these questions, various phase II or III trials are being conducted: TheraP (NCT03392428); PSMAfore (NCT04689828); UpFrontPSMA (NCT04343885); LuTectomy (NCT04430192); SPLASH (NCT04647526); and ECLIPSE (NCT05204927) are the most important ones.

Among these studies, TheraP (NCT03392428), an Australian phase II trial comparing ^177^Lu-PSMA-617 monotherapy and cabazitaxel (a semi-synthetic taxane) in patients with mCRPC and prior docetaxel treatment, is the only study that has been concluded, and has shown a more frequent PSA response in the ^177^Lu-PSMA-617 group (66% versus 37%, *p* < 0.0001), but a similar OS between the two treatment groups [12]. The remaining trials are expected to report initial data in 2023/24.

To increase the efficacy of RLT, several trials are testing combinations of ^177^Lu-PSMA-617 with other approved therapeutic agents. PSMAddition (NCT04720157) and ENZA-p (NCT04419402) will evaluate the efficacy of ^177^Lu-PSMA-617 associated with androgen receptor pathway inhibitors (ARPIs) versus ARPIs alone, while the LuPARP (NCT03874884) study will evaluate the safety and tolerability of olaparib in combination with ^177^Lu-PSMA-617. Another two trials (PRINCE, NCT03658447 and EVOLUTION, NCT05150236) will test the efficacy of ^177^Lu-PSMA-617 together with immune checkpoint inhibitors (pembrolizumab, ipilimumab, and nivolumab).

Other studies, such as the NCT04876651, are testing the efficacy of lutetium-177 conjugated to the monoclonal antibodies J591 or TLX-591 [13]. 

The rationale behind this last study is that monoclonal antibodies show less penetration into tissues, such as salivary glands and kidneys, compared to small ligands such as PSMA-617, and this may decrease the incidence of dry mouth and kidney damage, the two most frequent adverse effects seen in clinical trials [14].

Since one third of prostate cancer patients do not respond to lutetium-177, two prospective trials (AcTION, NCT04597411 and TATCIST, NCT05219500) have planned to test actinium-225, a radionuclide that delivers higher energy particles compared to the beta-particles of lutetium-177 [15]. These studies are enrolling patients both previously treated and untreated with lutetium-177, and will evaluate the efficacy and safety of ^225^Ac-PSMA-617 monotherapy.

Currently in progress, there is a phase I/II trial (NCT04886986) testing the combination of ^177^Lu-PSMA-617 with actinium-225 conjugated with the J591 monoclonal antibody [16] in patients with progressive mCRPC. The rationale behind this study is that the association of a monoclonal antibody/alpha-emitter with a small PSMA-ligand/beta-emitter may be synergistic in efficacy and show less adverse effects.

Lastly, an ongoing clinical trial (BAT-RAD, NCT04704505) is testing the efficacy of the combined treatment of the bone-targeted alpha emitter radium-223, and bipolar androgen therapy (BAT), a novel approach to hormone treatment, characterized by the administration of high-dose testosterone in patients receiving concurrent androgen deprivation therapy, which results in cyclic oscillations between supraphysiologic and near-castrate serum testosterone levels [17].

In conclusion, despite the heterogeneity and limitations of data, ^177^Lu-PSMA-617 RLT represents a safe and effective treatment for patients with mCRPC that has progressed after standard therapies. In addition, due to its low toxicity, it is an ideal therapeutic option for patients that do not tolerate other treatments or have extensive bone marrow involvement.

### 2.2. DNA Repair Inhibitors

DNA damage to cancer or normal cells is due to endogenous and exogenous events, such as the production of reactive oxygen species, or exposure to radiation or chemotherapy [18]. Depending on the type of genomic alteration [19], a specific DNA damage response (DDR) can be activated. Mismatch repair (MMR), base excision repair, or nucleotide excision repair pathways repair single nucleotide damage, whereas homologous recombination (HR) or non-homologous end joining pathways repair double stranded breaks (DSB) in DNA. Failure of the repair process triggers apoptosis as a response to genetic instability [20].

**Figure 2:**
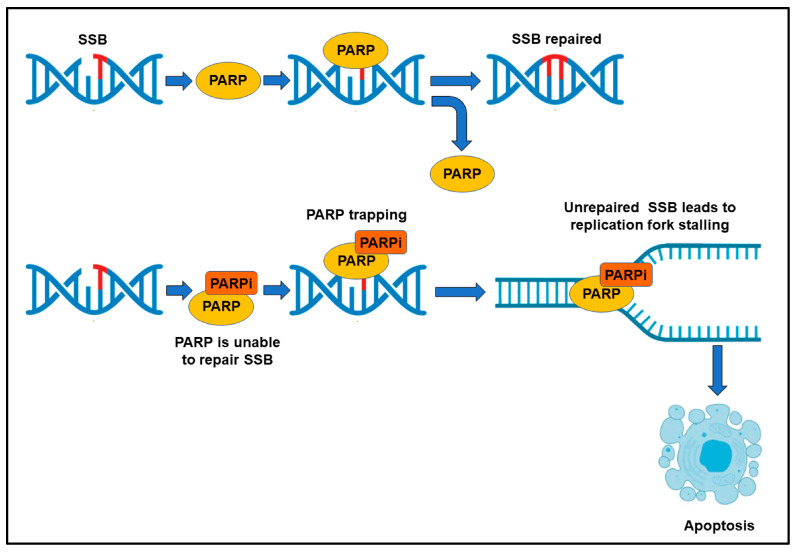
Mechanism of action of PARP inhibitors (PARPi). SSB: single strand break.

A high frequency of mutations promotes tumor progression, and genes involved in DSB repair, such as *BRCA1* and *BRCA2*, are frequently mutated in metastatic prostate cancers [21]. Somatic mutations have been detected in 20–25% of metastatic prostate cancer patients [22,23], whereas germline mutations in HR DNA repair genes involve 10–15% of these patients. *BRCA2* mutations are the most frequent (12–18%), followed by mutations of *ATM* (3–6%), *CHEK2* (2–5%), and *BRCA1* (<2%) genes [24,25]. This defective HR is partially compensated for by the hyperactivation of other DNA repair pathways, mediated by poly enzyme ADP-ribose polymerase 1, PARP1, and PARP2 [26]. After intercepting either single-strand or double-strand DNA breaks, PARP is enzymatically activated and polymerizes long chains of poly(-ADP)-ribose (PAR) on itself and other nuclear acceptor proteins, by using NAD+, and drives the PARylation process. The DNA repair machinery is recruited by PAR chains to the sites of DNA alteration (Figure 2) [27,28,29]. Frequently, the genomic integrity, and thus the survival of cancer cells to DNA damage, heavily depends on PARPs, and therefore PARP inhibitors (PARPi) [30] can dramatically affect cancer cell viability (synthetic lethality) (Figure 2).

In the last few years, two PARPi, olaparib and rucaparib, have been approved for the treatment of mCPRC [31].

Olaparib was approved by the Food and Drug Administration (FDA) in, May 2020, for the treatment of mCRPC with progression, after a second-generation hormonal agent (abiraterone or enzalutamide), in patients with mutations in any HR gene. It has been approved based on the data from a randomized phase III trial, the PROFOUND study, which showed a response ratio (33% vs. 2%) and an OS (19.1 months vs. 14.7 months) that were significantly higher in olaparib-treated patients compared to control patients, treated with only hormonal agents.

Since there are still unanswered questions regarding PARPi efficacy in hormone-sensitive prostate cancer; an ongoing study is testing the use of olaparib in men with biochemically recurrent prostate cancer following prostatectomy, without concurrent androgen deprivation therapy, and interim results show that 35% of olaparib-treated patients have a PSA response [32].

Rucaparib was approved by the FDA in 2020, after the conclusion of the TRITON2 study, which demonstrated that patients with *BRCA1* or *BRCA2* mutations, who had previously received both a second-generation hormonal agent and taxane-based therapy, and were then treated with rucaparib, had a PSA response rate of 54.8% [33].

There are two studies, currently active, on rucaparib: TRIUMPH (NCT03413995), assessing the efficacy of rucaparib in metastatic hormone-sensitive prostate cancer patients [34]; and ROAR (NCT03533946), determining the efficacy of rucaparib in patients with nonmetastatic, biochemically recurrent prostate cancer, after prostatectomy or radiation therapy.

Among the new PARPi, the most promising is talazoparib, which, in addition to strongly inhibiting the activity of catalytic enzymes, has revealed greater potency in trapping PARP1 to DNA errors [35]. PARP trapping indicates the increase in the binding affinity of PARP1 to damaged DNA, induced by PARPi. In brief, the PARPi blocks PARylation, and PARP1 remains tightly bound to damaged DNA [36]. As a result of PARP trapping, DNA replication is blocked; thus, the damage remains unrepaired and cell death occurs (Figure 2) [37].

An open-label phase II trial (TALAPRO-1) was carried out to test the efficacy of talazoparib in patients with mCRPC and homologous recombination repair (HRR) alterations [38]. The clinical response rate was 29.8% and reached 46% in patients bearing *BRCA1/2* mutations.

Since resistance to PARPi has been observed in the majority of patients with advanced tumors [39], various ongoing studies are focused on PARPi combinations, and the combination of PARPi with anti-androgen therapy, based on preclinical data that have demonstrated synergism between these two groups of therapeutic agents.

Interestingly, PARP promotes androgen receptor transcription; therefore, PARPi potentiate the effect of androgen deprivation therapy [40], which in turn promotes PARP overexpression, improving the response to PARPi, and boosts the expression of genes of the DDR pathway, leading to genomic instability and mutations, which favor sensitivity to PARPi. This mechanism is named BRCAness phenotype [41,42,43].

A 2018, phase II double-blind study [44] demonstrated a significant increase in progression-free survival in patients receiving a combination of PARPi with androgen receptor blockade. 

Recently, the MAGNITUDE phase III trial (NCT03748641) assessed the efficacy of niraparib with abiraterone acetate and prednisone (AAP) as a first-line therapy in mCRPC patients. Niraparib and AAP significantly improved progression-free survival in patients with *BRCA1/2* mutations, and reduced mortality by 47%. Combined treatment with niraparib and AAP was ineffective in patients without mutations in HR genes [45].

The phase III double-blind PROpel trial showed that the addition of olaparib to abiraterone reduced the risk for radiographic progression (up to 34%) in both in patients with and without HR gene mutations.

These results make olaparib the PARPi of choice for the first-line treatment of mCRPC.

Besides olaparib and talazoparib, the new PARPi veliparib is gaining great attention, since the NCT01576172 clinical trial showed an increase in the progression-free survival of patients treated with veliparib, administered in combination with abiraterone acetate and prednisone [46]. These positive results obtained by combining PARPi with anti-androgen therapy, have encouraged the development of new clinical trials investigating the combination of PARPi with other therapeutic strategies, such as immune checkpoint inhibitors, anti-VEGF therapies, AKT inhibitors/ATR inhibitors, and radionuclides. Definitive results from these studies will be announced in the coming years.

### 2.3. Therapies Targeting Tumor Neovascularization

Tumors need neovascularization to provide nutrition for their rapid growth and to discharge metabolic waste. Tumor vascular targeted therapy has been, since the 1970s, one of the most important research fields in oncology.

Vascular targeted therapy is based on targeting specific molecules expressed by tumor endothelial cells. The targeting of these molecules can inhibit angiogenesis and tumor growth. Vascular endothelial growth factor (VEGF) and endothelin (ET) are the main targets of anti-angiogenic therapies, most of which have already entered clinical trials.

VEGF is the most important regulatory cytokine in tumor angiogenesis. It is highly expressed in most cancer cells, but it is also produced by fibroblasts, and the endothelial and immune cells of the tumor microenvironment [47]. The biological effect of VEGF on endothelial cells is mediated by two receptor tyrosine kinases (RTKs), VEGFR-1 (Flt-1) and VEGFR-2 (KDR or Flk-1), with different signaling properties, which can be modulated by non-signaling co-receptors [48]. Among VEGF isoforms, VEGF-A is overexpressed in prostate cancer, by cancer cells, endothelial cells, and stromal fibroblasts, and has been demonstrated to play a key role in prostate cancer angiogenesis and progression [49]. High levels of VEGF-A have been associated with distant metastasis and poorer prognosis [50,51,52,53].

These findings have led to the clinical development of a variety of VEGF inhibitors, which have been tested for the treatment of advanced prostate cancer.

A randomized, double-blind, placebo-controlled phase III clinical study (NCT00110214), which enrolled 1050 patients, not only failed to improve overall survival in mCRPC when bevacizumab (humanized anti-VEGF monoclonal antibody) was used together with docetaxel chemotherapy and prednisone hormonal therapy, but also revealed that the administration of bevacizumab alone caused side effects and treatment-related deaths [54]. This result suggests that in mCRPC, in which conventional treatments are often ineffective, the addition of bevacizumab to standard therapies has no beneficial effects.

Other molecules targeting the VEGF-A pathway (e.g., aflibercept and sunitinib) were tested in large clinical trials (NCT00676650, 873 patients, and NCT00519285, 1224 patients) and have proven ineffective in the treatment of mCRPC [55,56]. Furthermore, even when the administration of anti-angiogenic agents has led to a slight improvement in overall survival, they have been associated with an increased toxicity rate and adverse effects (fatigue, asthenia, pulmonary embolism, hypertension, peripheral blood cytopenia, intestinal perforation and/or bleeding), forcing the discontinuation of treatment [56,57]. 

Altogether, these results suggest that the combination of anti-angiogenic therapy with chemotherapy or hormonal therapy in mCRPC has no beneficial effects. The redundancy of angiogenic pathways could be one of the possible explanations for the lack of a therapeutic response, since targeting a single pathway may be compensated by the upregulation of alternative pathways. Therefore, it is conceivable that targeting these alternative pathways could provide an effective anti-angiogenic therapy. A phase II study, demonstrated that in 63 patients with mCRPC, in which bevacizumab was administered in combination with the immunomodulatory drug lenalidomide (a 4-amino-glutamyl analogue of thalidomide, with potent antineoplastic, anti-angiogenic, and anti-inflammatory properties), docetaxel, prednisone and hormonal therapy, anti-angiogenic therapy can be safely combined with other therapeutic approaches, but further clinical trials are required to confirm this result [58].

ET is a polypeptide composed of 21 amino acids and its main function is to regulate cardiovascular tension. It has been found that ET and its receptors are expressed in advanced prostate cancer with bone metastasis [59,60], and Sugawara et al. found that ET receptor antagonists, combined with androgen deprivation, can significantly reduce bone metastases [61]. Therefore, the development of ET targeted therapy, which is currently at the experimental stage, is eagerly awaited for the treatment of advanced prostate cancer.

### 2.4. Immune Checkpoint Inhibitors

Since prostate cancer is characterized by a low tumor mutational burden, low expression of programmed death-ligand 1 (PD-L1), and scarce T-cell infiltration, it has been considered refractory to immunotherapy. Furthermore, the clinical trials carried out, to date, have not shown encouraging results.

In a phase I trial, only 2 out of 14 patients with mCRPC showed PSA decreases of ≥ 50% after receiving an anti-CTLA-4 antibody [62]. 

In another phase I trial, using a humanized anti-CTLA-4 antibody, the PSA doubling time was prolonged only in 3 out of 11 patients [63].

In a phase I/II study, an anti-CTLA-4 antibody, alone or in combination with radiation, was given to 50 patients with mCRPC. Only six patients had stable disease, one had a full response, and eight had PSA decreases of less than 50% [64].

Anti-CTLA-4 and anti-PD-1 antibodies were administered in combination to patients with mCRPC, in a phase II clinical trial, achieving a 25% objective response rate (ORR), but the treatment caused substantial adverse effects [65].

Two phase III studies (NCT01057810 and NCT00861614), which recruited 837 and 799 patients, respectively, tested the efficacy of ipilimumab vs. placebo in mCRPC and found no association between drug administration and overall survival [66,67]. In another large phase III study (NCT03016312), which recruited 759 patients, the addition of atezolizumab to enzalutamide did not show any increase in overall survival compared to enzalutamide alone [68].

Only one phase III trial showed a significant progression-free survival increase in mCRPC patients treated with radiation, followed by anti-CTLA-4 antibody [67,69,70].

Despite their successes in treating a variety of advanced-stage cancers, poor clinical outcomes have been obtained with active immunotherapies in prostate cancer patients, due to a range of limitations, including the low level of targeting molecules and the consequences of long-term androgen deprivation therapy, which polarizes tumor infiltrating immune cells toward immunosuppression [71]. Various immunosuppressive mechanisms orchestrate the tumor microenvironment (TME) [72], which consist of immune and stromal cells, blood vessels, extracellular matrix components, and various signaling molecules [73,74] and contribute to the failure of immunotherapy in prostate cancer patients. Tumor-associated macrophages (TAM) are among the main culprits of the highly immunosuppressive prostate TME, representing 30% to 50% of the infiltrating immune cells. Together with myeloid-derived suppressor cells and T-regulatory cells, TAMs are recruited by chemokines and cytokines released by prostate cancer cells [72] and contribute to tumor immune escape, and anti-androgen and chemotherapy resistance, leading to tumor growth and progression [75]. Therefore, several preclinical investigations and clinical trials are currently active, aiming to assess the efficacy of different macrophage-targeting therapies in prostate cancer [76].

Ongoing strategies to target macrophages aim to reduce their migration and intra-tumoral recruitment, or to promote their death or depletion, or to reprogram their functions at the tumor site. CSF-1R inhibitor (JNJ-40346527), which inhibits macrophage recruitment and survival, and CAR-M (CT-0508) (NCT04660929), which redirects macrophage phagocytosis toward HER2^+^ cancer cells, are currently in phase I clinical trials, respectively, for the treatment of high-risk localized prostate cancer (NCT03177460) and for the treatment of HER2-overexpressing prostate cancer. It has been demonstrated that CXCR2 blockade re-educates TAMs and hinders prostate cancer [77]. Promising results have been obtained from the combined treatment of a CXCR2 antagonist, AZD5069, with an AR antagonist, enzalutamide, currently in a phase I/II clinical trial for the treatment of mCRPC (NCT03177187), and from the combined treatment of a CXCR1/2 antagonist, navarixin (MK-7123) with pembrolizumab (anti-PD-1), which has already passed a phase II trial (NCT03473925) [76].

Cancer-associated fibroblasts (CAFs) are the major cellular stromal component of the prostate TME. Activated by the crosstalk with cancer cells, CAFs release cytokines and growth factors, reprogram the extracellular matrix, contribute to the stem cell niche and resistance to chemotherapy, and promote cancer invasiveness and metastasis [78]. It has been demonstrated that most prostate CAFs express AR, and that the androgen-triggered AR/filamin A (FlnA)/b1 integrin complex regulates extracellular matrix remodeling and drives CAF migration and invasiveness, which can be inhibited by using an AR-derived stapled peptide that specifically prevents AR/FlnA complex assembly in androgen-treated CAFs [79]. Clinically approved drugs for the therapeutic targeting of CAFs are still lacking; however, several trials are ongoing to evaluate the antitumor efficacy of drugs targeting cancer-promoting CAF functions [80,81,82]. A role in regulating the crosstalk among CAFs, prostate cancer cells, and other cells of the TME has recently emerged for nerve growth factor (NGF). NGF is a neurotrophin (NTR) family member, along with the prototypic NT, brain-derived neurotrophic factor (BDNF), neurotrophin-3 (NT-3), and neurotrophin-4/5 (NT-4/5). NGF/TrkA, NT-4/5/TrkB, and BDNF/TrkB axes may not only stimulate prostate cancer cell proliferation and metastasis, but may also promote perineural invasion and associated pain, and, therefore, have been identified as significant therapeutic targets [83,84,85]. To our knowledge, therapeutic strategies targeting NTR-regulated signaling pathways are currently in the preclinical stages of development.

The last, but no less relevant, cause of the poor success of immunotherapy for the treatment of prostate cancer lies in the genetic heterogeneity and multifocality of this type of cancer, resulting in the multiplicity of tumor clones, each having a different degree of differentiation, genomic alterations, and transcriptional and antigenic profiles [86,87,88], which make them differentially susceptible to immunotherapy. 

In a future perspective, a careful selection of patients according to biological markers may improve the clinical response to immunotherapy. For example, tumors with microsatellite instability (MSI) and deficient MMR enzymes, which are characterized by an accumulation of a mutational burden, a potential source of immunogenic neoantigens leading to a high immunoscore and cytotoxic T cell infiltration [89], are more responsive to immune checkpoint blockade. Although MMR/MSI status could serve as a powerful and reliable biomarker for the optimization of patient selection for immunotherapy and the prediction of responsiveness and prognosis [89,90], its frequency is very low in prostate cancer and involves only 3–8% of patients. This could explain the scarce T cell infiltration observed in the prostate TME. The intra-tumoral T cell infiltrate, and the whole immune cell context, can be shaped by a patient’s co-morbidities, such as metabolic, or chronic inflammatory, or infectious diseases, such as those caused by SARS-CoV-2, COVID-19, which suppress both innate and adaptive immune responses leading to a long-lasting decrease in CD4^+^ and CD8^+^ T lymphocytes [91,92].

Importantly, most of the patients diagnosed with prostate cancer are affected by co-morbidities, as they are elderly. The ageing immune system provides a weak immune response [93], and the efficacy of immunotherapy relies on an efficient immune system.

## 3. Future Challenges

The efficacy of molecular targeted therapies relies on their ability to selectively hinder the signaling pathways that regulate tumor cell proliferation, survival and/or progression. 

The development of these new therapies, which have changed the therapeutic paradigm of prostate cancer, raises the questions of how to combine these therapeutic agents with classical treatment approaches, such as chemotherapy and androgen deprivation therapy, and what the most effective administration sequence of these drugs is. Recent studies have demonstrated that in patients with androgen-independent metastatic prostate cancer, the most promising results were achieved when combining a targeted therapy with standard therapy strategies. For example, the Akt inhibitor ipatasertib re-sensitized androgen-independent cells to antiandrogens, and, when combined with the androgen receptor antagonist enzalutamide, led to remarkable tumor cell growth inhibition, both in vitro and in vivo [94]. The glucocorticoid antagonist RU486 [95] and the PI3K/mTOR inhibitor BEZ235 [96] were also reported to re-sensitize resistant cancer cells to standard chemotherapeutic agents. Lastly, the combination of standard radiation therapy and PARPi demonstrated a significant effect on tumor progression, since veliparib [97] and rucaparib [98] were shown to re-sensitize androgen-independent prostate cancer cells to radiotherapy, impairing tumor growth.

Although most of the molecular targeted treatments have been proven effective in the management of mCRPC, resistance frequently occurs, as in the case of the PI3K inhibitor CUDC-907 [99] and the ERK inhibitor PD325901 [100], which by triggering compensatory signaling mechanisms in tumor cells demonstrated reduced therapeutic efficacy. The development of combined therapies could overcome drug resistance and improve clinical and pathological responses.

Among the most promising molecular targeted agents currently under study [101,102], there are drugs targeting DNA methylation and demethylation.

Two DNA methyltransferase (DNMT) inhibitors, decitabine and azacytidine, have been approved by the FDA for clinical use in the treatment of myelodysplastic syndrome and acute myeloid leukemia [103]. After their incorporation into DNA, DNMT inhibitors have extensively reduced methylation levels [104]. Histone acetyltransferases [105], histone deacetylases [106,107], histone demethylases [108], histone methyltransferases [109], and DNA methyltransferase inhibitors [110] are currently used in preclinical studies and clinical trials for the treatment of mCRPC, and demonstrate anti-tumoral effects mostly due to gene expression reprogramming. The best results were obtained when these agents were combined with standard androgen derivation therapy [111], docetaxel [112], radiation therapy [113], or other drugs targeting the epigenetic machinery [108,114].

The most innovative approach to the molecular targeting of cancer, including prostate cancer, is nanomedicine-based strategies, which aim to enhance drug delivery, improve treatment efficacy, and minimize side effects. Nanoparticles of different formulations, such as liposomes, polymeric nanoparticles, and inorganic nanoparticles, can be designed to encapsulate molecular targeted drugs, and to selectively accumulate in prostate tumors through conjugation with antibodies that specifically recognize tumor-associated markers, such as PSMA, enhancing tumor targeting and internalization. Liposomes loaded with doxorubicin (Doxil) or micelles carrying paclitaxel (Apealea, Genexol-PM) have already been approved for clinical use [115], whereas the nanoparticle-mediated delivery of molecular targeted drugs is currently still being tested. Notably, nanoparticles can deliver therapeutic genes to both prostate cancer cells and cells of the TME. For instance, polyplex micelles based on the self-assembly of PEG20kDa-poly{N0-[N-(2-aminoethyl)-2-aminoehtyl]aspartamide}-cholesteryl [PEGPAsp(DET)-cholesteryl] (PEG20C), and plasmid DNA encoding soluble vascular endothelial growth factor receptor-1 (sVEGFR-1) or soluble fms-like tyrosine kinase-1, sFlt-1 (endowed with antiangiogenic property), conjugated with cRGD peptide ligands, selectively targeting ⍺vβ5 and ⍺vβ3 integrin receptors [116], which are greatly expressed on cancer cells and tumor vascular endothelial cells [117,118,119,120]. Encouraging results have been achieved with the systemic administration of this micellar formulation in models of pancreatic cancer, which have a robust stromal component similar to prostate cancer, in which it is expected to be tested. 

Lastly, since these innovative therapeutic agents entered clinical practice only a few years ago, data regarding their long-term effects are not yet available. Thus, it will be essential to carefully evaluate the impact of these therapies on the health status of long-surviving patients, who often present with other comorbidities.

## 4. Conclusions

The management of mCRPC has been deeply modified by the introduction of a variety of new therapies, such as PSMA-targeted radionuclide treatments, PARPi, and molecules targeting tumor neovascularization and immunotherapy.

The new treatment paradigm of mCRPC, based on molecular targeting approaches and the discovery of novel molecular biomarkers of outcome prediction, requires accurate patient selection, based on the patient’s clinicopathological characteristics and tumor molecular profiles; this allows for an estimation of the efficacy outcomes of the different molecular targeted agents [121], and the design of the correct drug sequence for combination treatments, reducing side effects and improving survival.

This overview on molecular targeted therapies, which describes both experimental studies and ongoing clinical trials, as well as drugs already approved for clinical use, summarizes the wide range of therapeutic options for the different clinical and pathological stages of the disease. However, it also highlights the critical issues to be overcome by proposing solutions, and, with respect to what has been described so far [101,122,123], illustrates possible ways forward to enhance effectiveness and to personalize molecular targeted drugs to overcome side effects and improve life expectancy.

## Figures and Tables

**Table 1:** Molecular targeted agents for mCRPC treatment.

Therapeutic Agent	Mechanism of Action
	
**PSMA-targeted radionuclides**	
^177^Lu-PSMA-617	Beta-emitting radioisotope Lutetium-177 conjugated with the PSMA ligand PSMA-617
^177^Lu-DOTA-rosopatamab	Lutetium-177 conjugated, via the chelating agent dodecanetetraacetic acid (DOTA), with rosopatamab, a humanized monoclonal antibody against PSMA
^225^Ac-PSMA-617	Alpha-emitting radioisotope Actinium-225 conjugated with PSMA-617
^225^Ac-PSMA-I&T	Actinium-225 conjugated with the PSMA ligand PSMA-I&T
	
**DNA repair inhibitors**	
Olaparib	Inhibitor of PARP1 and PARP2
Rucaparib	Inhibitor of PARP1, PARP2 and PARP3
Talazoparib	Inhibitor of PARP1 and PARP2
	
**Therapies targeting tumor neovascularization**	
Bevacizumab	Monoclonal antibody specific for VEGF-A
Aflibercept	Inhibitor of VEGF-A and -B
Cediranib	Inhibitor of VEGFR-1, -2 and -3
Sunitinib	Inhibitor of VEGFR-1, -2, -3; PDGFR-A and -B
	
**Immune checkpoint inhibitors**	
Atezolizumab	Monoclonal antibody specific for PD-L1
Ipilimumab	Monoclonal antibody specific for CTLA-4
Pembrolizumab	Monoclonal antibody specific for PD-1
	

CTLA-4: cytotoxic T-lymphocyte antigen 4. PD-1: programmed cell death protein 1. PD-L1: programmed death-ligand 1.

**Table 2:** Main clinical trials testing molecular targeted agents in mCRPC.

Therapeutic Agent	Clinical Trial N. (Acronym)	Patients Eligibility Criteria	Outcomes
			
^177^Lu-PSMA-617	NCT03511664 (VISION)	✓ PSMA^+^mCRPC ✓ Prior systemic therapy with − androgen receptor inhibitors − taxane chemotherapy	Improvement in overall survival
^177^Lu-DOTA-rosopatamab	NCT04876651 (PROSTACT)	✓ PSMA^+^mCRPC ✓ Prior systemic therapy with − one novel androgen axis drug	Ongoing. Data not yet available
^225^Ac-PSMA-617	NCT04597411 (AcTION)	✓ PSMA^+^mCRPC	Ongoing. Data not yet available
^225^Ac-PSMA-I&T	NCT05219500 (TATCIST)	✓ PSMA^+^mCRPC	Ongoing. Data not yet available
Olaparib	NCT02987543 (PROFOUND)	✓ mCRPC ✓ Mutations in any HR gene ✓ Prior systemic therapy with − abiraterone or enzalutamide	Improvement in overall survival
Rucaparib	NCT02952534 (TRITON2)	✓ mCRPC ✓ BRCA1 or BRCA2 mutations ✓ Prior systemic therapy with − enzalutamide, apalutamide or darolutamide − taxane chemotherapy	Improvement in PSA response rate
		
	NCT03413995 (TRIUMPH)	✓ Metastatic hormone-sensitive PC ✓ Mutations in any HR gene ✓ No prior androgen deprivation therapy	Ongoing. Data not yet available
		
	NCT03533946 (ROAR)	✓ Nonmetastatic hormone-sensitive PC ✓ BRCAness * ✓ No prior treatment with PARP inhibitor or platinum based chemotherapy	Ongoing. Data not yet available
Talazoparib	NCT03148795 (TALAPRO-1)	✓ mCRPC ✓ Mutations in any HR gene ✓ Prior systemic therapy with − taxanes − enzalutamide and/or abiraterone acetate/prednisone	Improvement in radiolgical response rate
Ipilimumab	NCT01057810	✓ mCRPC ✓ Prior androgen deprivation therapy	No improvement in overall survival
	NCT00861614	✓ mCRPC ✓ Prior treatment with docetaxel	No improvement in overall survival
			

* BRCAness: alteration in one or more of the following genes, *BARD1*, *BRCA1*, *BRCA2*, *BRIP1*, *CHEK1*, *CHEK2*, *FANCA*, *NBN*, *PALB2*, *RAD51C*, *RAD51D*, *RAD51*, *RAD51B*.

**Table 3:** Main clinical trials testing the efficacy of different combinations of molecular targeted agents and standard-of-care therapies for the treatment of mCRPC.

Combination Tested	Clinical Trial N. (Acronym)	Patients Eligibility Criteria	Outcomes
			
^177^Lu-PSMA-617 + androgen deprivation therapy	NCT04720157 (PSMAddition)	✓ mCRPC	Ongoing. Data not yet available
^177^Lu-PSMA-617 + enzalutamide	NCT04419402 (ENZA-p)	✓ mCRPC ✓ No prior treatment with docetaxel	Ongoing. Data not yet available
^177^Lu-PSMA-617 + olaparib	NCT03874884 (LuPARP)	✓ mCRPC ✓ Disease progression after androgen receptor-targeted therapy (e.g., enzalutamide, abiraterone acetate, apalutamide)	Ongoing. Data not yet available
^177^Lu-PSMA-617 + pembrolizumab	NCT03658447 (PRINCE)	✓ mCRPC ✓ Disease progression after androgen receptor-targeted therapy (e.g., enzalutamide, abiraterone acetate, apalutamide)	Ongoing. Data not yet available
^177^Lu-PSMA-617 + ipilimumab and nivolumab	NCT05150236 (EVOLUTION)	✓ mCRPC ✓ Disease progression after androgen receptor-targeted therapy (e.g., enzalutamide, abiraterone acetate, apalutamide)	Ongoing. Data not yet available
^177^Lu-PSMA-I&T + ^225^Ac-J591 *	NCT04886986	✓ mCRPC ✓ Disease progression after androgen receptor-targeted therapy (e.g., enzalutamide, abiraterone acetate, apalutamide)	Ongoing. Data not yet available
^223^Radium + bipolar androgen therapy (BAT)	NCT04704505 (BAT-RAD)	✓ mCRPC ✓ Prior treatment with gonadotropin-releasing hormone analogues or bilateral orchidectomy	Ongoing. Data not yet available
Niraparib + abiraterone acetate + prednisone	NCT03748641 (MAGNITUDE)	✓ mCRPC ✓ Mutations in any HR gene	Improvement in progression-free survival in patients with alterations in HR genes
Olaparib + abiraterone acetate	NCT01972217 (PROpel)	✓ mCRPC ✓ No prior chemotherapy, PARP inhibitor administration or androgen receptor-targeted therapy.	Reduction of radiographic progression of 34%
Olaparib + cediranib	NCT02893917	✓ mCRPC ✓ Prior chemotherapy	Improvement in progression-free survival compared with olaparib alone
Veliparib + abiraterone acetate + prednisone	NCT01576172	✓ mCRPC ✓ Prior androgen deprivation therapy	Improvement in PFS
Atezolizumab + enzalutamide	NCT03016312	✓ mCRPC ✓ Prior androgen synthesis inhibitor therapy	No improvement in overall survival
Bevacizumab + docetaxel + prednisone	NCT00110214	✓ mCRPC ✓ No prior cytotoxic chemotherapy or anti-angiogenic therapy	No improvement in overall survival
Sunitinib + prednisone	NCT00676650 (SUN 1120)	✓ mCRPC ✓ Prior treatment with docetaxel	No improvement in overall survival
Aflibercept + docetaxel + prednisone	NCT00519285 (VENICE)	✓ mCRPC ✓ Prior treatment with VEGF or VEGFR inhibitors	No improvement in overall survival
			

* J591: monoclonal antibody specific for PSMA.

## Data Availability

No new data were created or analyzed in this study. Data sharing is not applicable to this article.
